# Supplementary material for: The mechanism underlying redundant functions of the YTHDF proteins
Source: Genome Biol. 2023 Jan 24;24:17. doi: 10.1186/s13059-023-02862-8 (PMC9872407; doi:10.1186/s13059-023-02862-8)
Supplement: Supplementary file 1 — Additional file 1: Figure S1 describes discrepancies between experimental results and the unified model of YTHDFs. Figure S2 illustrates increased P-body formation after YTHDF1-3 triple knockdown. Figure S3 contains uncropped gel images shown in the figures. [file 13059_2023_2862_MOESM1_ESM.docx]

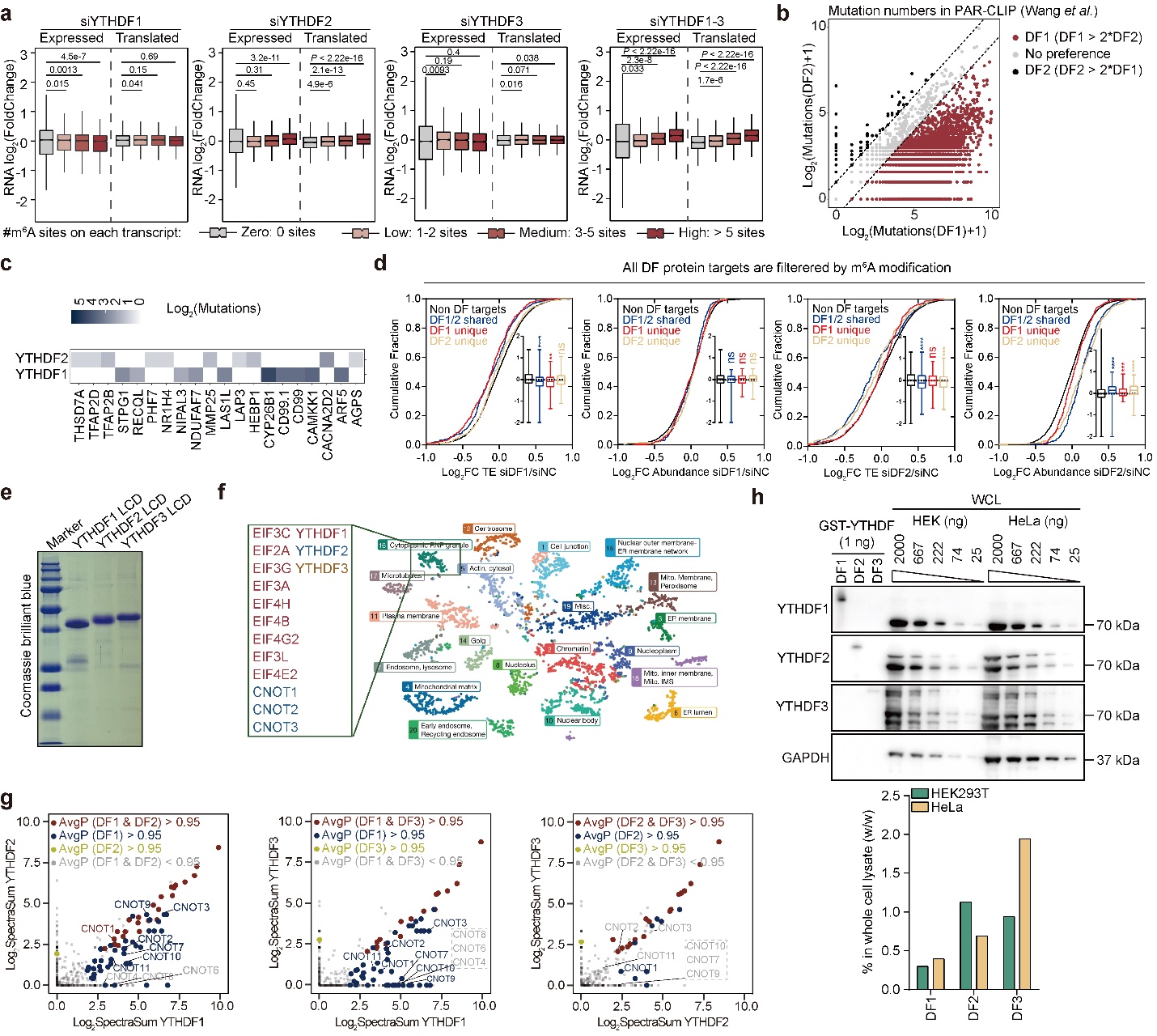


**Fig. S1 Discrepancies between experimental results and the unified model of YTHDFs**

1. Boxplots showing fold changes of transcripts with different numbers of m^6^A sites when analyzing all expressed genes (“Expressed”) or only translated genes (“Translated”) after individual knockdown of YTHDFs. Transcripts were classified by individual numbers of m^6^A sites (zero: 0 sites, low: 1-2 sites, medium: 3-5 sites, high: more than 5 sites). For boxplots, the center line represents the median, the box limits show the upper and lower quartiles, whiskers represent 1.5 × interquartile range. *P* values were determined by a Mann-Whitney-Wilcoxon test.
2. Scatter plot showing T-C mutations of each gene in YTHDF1 and YTHDF2 PAR-CLIP experiments. Red dots represent genes with DF1 mutation numbers more than 2 × DF2 mutation numbers. Black dots represent genes with DF2 mutation numbers more than 2 × DF1 mutation numbers. Mutation frequencies were summarized with omniCLIP.
3. Heatmap showing mutation numbers on selected transcripts specifically bound by YTHDF1 or YTHDF2.
4. Cumulative plots showing changes in RNA translation efficiency (TE) and abundance after *YTHDF1* (left) or *YTHDF2* (right) knockdown using data from Zaccara *et al.* *P* values were determined by a Mann-Whitney-Wilcoxon test. (*, *P* < 0.05; **, *P* < 0.01; **, *P* < 0.001; ****, *P* < 0.0001; *ns*, *P* > 0.05)
5. SDS-PAGE analysis of purified YTHDF protein LCDs.
6. Demonstration of the original protein localization map of the cell generated by t-distributed stochastic neighbour embedding (t-SNE) from the CELL MAP project. The cluster annotated as cytoplasmic RNP granules containing YTHDF proteins was selected for analysis in Fig. 1g.
7. Scatter plots showing comparisons of YTHDF protein interactions between YTHDF1/YTHDF2 (left), YTHDF1/ YTHDF3 (middle), and YTHDF2/YTHDF3 (right). Processed Bio-ID results were obtained from Youn *et al*. All comparisons were made using data for C-terminal BirA* fusion proteins.
8. Top: Western blots showing relative protein levels of YTHDFs in WT HeLa and HEK293T cells. Recombinant GST tagged YTHDF proteins were used as loading controls. Bottom: quantification of western blots using ImageJ.


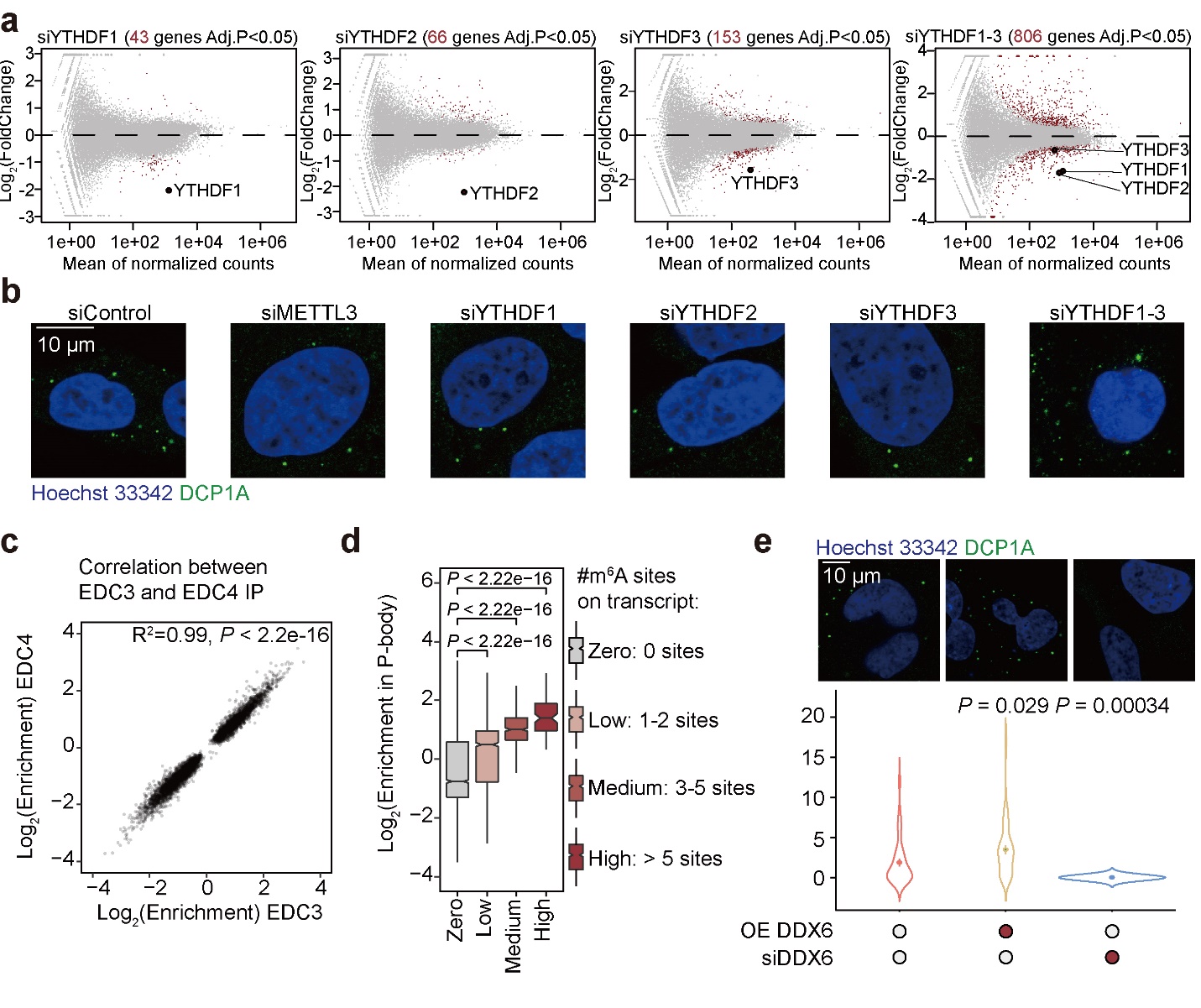


**Fig. S2 Increased P-body formation after YTHDF1-3 triple knockdown.**

1. MA plots showing RNA expression changes after single knockdown or triple knockdown of YTHDFs. Grey dots denote genes not significantly altered and red dots denote significantly differentially expressed genes. Genes targeted by siRNAs are labeled.
2. Representative images from P-body imaging in HeLa cells, related to figure 2b. P-bodies were stained using a DCP1A antibody and cell nuclei were counterstained by Hoechst 33342. Eight images were captured for each condition in n = 3 individual experiments.
3. Scatter plot showing correlation between RNA enrichments in P-body transcriptomes determined with EDC3 or EDC4 antibodies. *P* value was determined by Pearson’s correlation test.
4. Boxplots showing enrichments of different transcripts grouped by numbers of m^6^A sites identified by MeRIP-seq (zero: 0 sites, low: 1-2 sites, medium: 3-5 sites, high: more than 5 sites). For boxplots, the center line represents the median, the box limits show the upper and lower quartiles, whiskers represent 1.5 × interquartile range. *P* values were determined by a Mann-Whitney-Wilcoxon test.
5. Fluorescence microscopy analysis of P-body numbers after overexpression (OE) or knockdown of DDX6 in HeLa cells. Numbers of DCP1A foci per cell were quantified with CellProfiler 3.0. *P* values were determined by a *t* test. Number of cells analyzed per condition: control (30), OE DDX6 (23), siDDX6 (45).

**
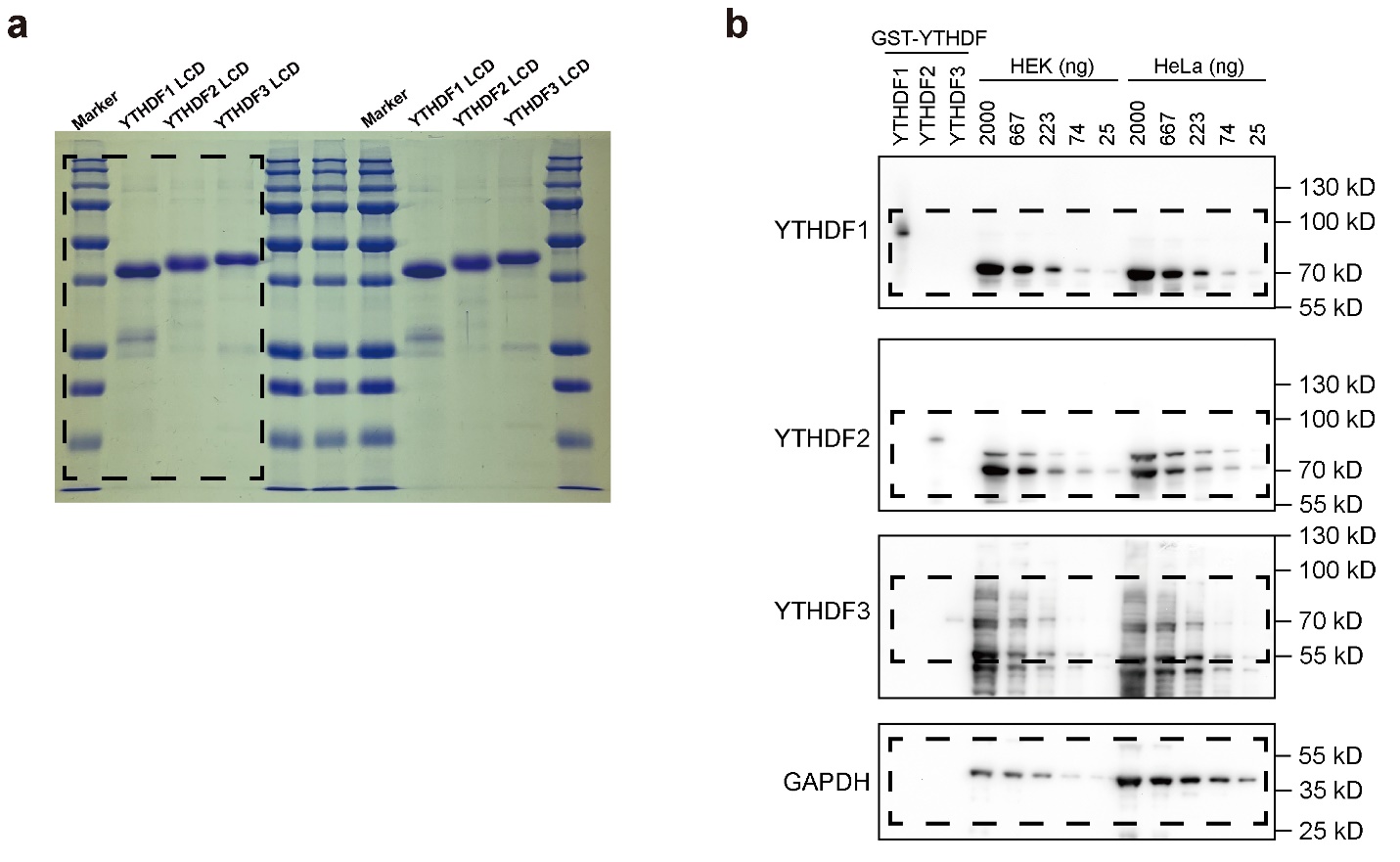
**

**Fig. S3 Uncropped gel images.**

1. Uncropped gel image for Fig. S1e.
2. Uncropped western blot images for Fig. S1h.
