# Supplementary material for: The mechanism underlying redundant functions of the YTHDF proteins
Source: Genome Biol. 2023 Jan 24;24:17. doi: 10.1186/s13059-023-02862-8 (PMC9872407; doi:10.1186/s13059-023-02862-8)
Supplement: Supplementary file 3 — Additional file 3. Review history. [file 13059_2023_2862_MOESM3_ESM.docx]

**Review history**

**First round of review**

**Reviewer 1**

The YTHDF m6A reader proteins (YTHDF1, 2, and 3) have all been shown previously to bind m6A-containing mRNA. While each protein has been shown to have different effects on regulating RNA biology, including translation for DF1 and RNA decay for DF2, and on specific phenotypes (e.g. in KO mouse models), a recent manuscript (Zaccara & Jaffrey, PMID: 32492408) has suggested that the three proteins are redundant for each other and that they only regulate mRNA degradation and cellular differentiation. This was based in part on the finding that depletion of three results in increased mRNA abundance, with no effects on translation. This manuscript attempts to show that in fact these proteins, specifically DF1 and DF2, do have specific, individual roles in RNA biology and they are not redundant. Intriguingly, the manuscript presents data that suggests that loss of all three of the DF proteins, but not individual, actually results in increased formation of P bodies where m6A RNA is enriched and stabilized, and that might explain previous observations that showed that loss of all three proteins phenocopies loss of DF2. While the study is systematic in re-analyzing previous data to show that YTHDF proteins do in fact have individual functions, there are instances where rationale was not clear and certain controls and interpretations needed to be included, revised, or explained more clearly (see comments below). However, the analyses do support a conclusion that YTHDF proteins have individual function to regulated specific subsets of RNA (YTHDF CLIP targets) and previous analyses of larger subsets of RNA (total or actively translated) obscured these functions. Thus, overall, this is an important study as it attempts to clarify the differences between published studies, and it will be important for the RNA biology field.

Comments:
* The text of this manuscript is written in a very antagonistic and judgmental way. See Line 55, "two flaws", Line 63, "not germane" Line 81, not appropriate; Line 84, "skewed choices". The manuscript would be more powerful if the work was presented in a non-judgemental fashion. Just say what was seen in the previous work, and then say what you did and why the results differ. For example, rather than saying "It is not germane to analyze the effects of YTHDF1 knockdown on translation or stabilization of ~60% of the transcriptome as potential targets of YTHDF1…" one could say, "While a previous study examined the effects of YTHDF1 knockdown on translation or stabilization of ~60% of the transcriptome…., as we know that the protein only binds ~10% of the transcriptome, we chose to specifically study the effects of YTHDF1 on its bound transcripts"… etc .
* In Figure 1, data is presented on an m6A target centric (which m6A-modified mRNAs are regulate by DF1/DF2) vs RBP target centric approach (only looking at RNAs known to be bound by DF proteins). While the text clearly stated the limitations of an m6A-only approach, it wasn't obvious why the analyses performed were not filtered for transcripts with both m6A and RBP peaks from sequencing (m6A and RBP target centric approach). This could be even more informative.
* In Fig. 1A-1B, the numbers in the Venn diagrams vs those described in the text were not always clear. It would be helpful to label the figures differently. For example,
♣ Fig. 1a: The text describes 4424 transcripts analyzed in Zaccara et al vs the 753 high confidence transcripts bound by YTHDF1, but it is unclear where this number comes from as the DF1 target numbers are 432+259+321=not 753.
♣ The overlaps and comparisons are difficult to follow in the text vs figure (referencing 7105 and 6814 when those are not in the diagrams without adding the numbers). Consider revising the text or figures (perhaps upset plots would help convey the points).
♣ Figure 1B doesn't really make sense and the pictures aren't helpful. Perhaps just make a venn diagram with a legend.
* The data showing that purified YTHDF2 protein might make fibrils is potentially interesting, however some control data is missing. For example, how pure are the protein preps of each? I didn't see a Coomassie gel, etc. If each one has slightly different purification purities, this could explain the results. Also, it seems like the same time course (fresh, 2 weeks, 4 weeks) should be showing for the other YTHDF proteins to aid in comparison. Finally, while I do think this could get at the fact that the different proteins have different "natures", I'm not sure how this is related at all to their function, and this should be addressed.
* For the data comparisons of the YTHDF proteomics shown in Figure 1G and Supplemental Figure 1B, it's unclear why this analysis led to a different result than previous analyses. A more detailed method for analyses of the publicly available proteomics data is required, as well as a discussion that compares the analyses methods used by this paper and the Zaccara paper and describes possible discrepancies. It's not helpful to the field to just say the other paper is wrong.
* The data shown in Figure S1d are not convincing enough to show for the relative abundances of these proteins between the cell lines tested, in particular because the loading control GAPDH is not even in the replicates. A total protein stain should be done as the loading control, and a linear range of protein should be used, with purified protein as a control (e.g. 5ug, 20ug, 50ug protein).
* The quantification of the number of p bodies under different conditions (see Supp Fig 2B) doesn't seem right. When I look at the representative figures, it appears that siDF1 has a large increase in the number of P bodies as compared to siMETTL3. Thus, its unclear how this data led to the results shown in Fig 2B. Further, without having a cytoplasmic marker (or bright-field) markers, one can't examine whether the green dot for the p body is in a cell or just background. Finally, it is really hard to see the p body staining, and some image processing that makes them more clear for the figures would be helpful.
* The last paragraph of the results was confusing and difficult to read, in particular the figure legends and data didn't always seem to match what was said in the text.
* While the conclusion that YTHDF1-3 dependent stabilization of RNA is P-body, not m6A, -dependent is supported, it is not clear if P-body formation is sufficient for the phenotype. The text does state "that the global stabilization of mRNA after depletion of all YTHDF proteins is a result of increased P-body formation and is not strictly m6A dependent," however, an important experiment to support this claim would be to induce P-body formation with a stimuli other that YTHDF knockdown and observe RNA stability, perhaps over-expression of DDX6?


Minor comments:
* In line 31 of background, YTHDF1 function is described, but no detail on known function of others. A short description/summary would be helpful.
* Fig. S1A: YTHDF3-dependent decreases are not obvious in these data. Text should be revied to only include YTHDF1-dependent changes
* Fig. S1A: YTHDF3-dependent changes in translated genes appear to be more significant than expressed genes alone (according to indicated stats). This is contrary to the point made in the text. Please fix figures if needed or revise test to match data.
* Fig 1C-D: To better understand the difference between the conclusion of the authors and previously held models, please include differences in TE and abundance upon YTHDF1 or 2 knockdown when the original set 4424 transcripts is analyzed rather than exclusively YTHDF1 or 2 targets (compare with m6A data)
* Line 106: Please described how the YTHDF proteins differ in their low-complexity domains, rather than saying they differ "greatly"; Similar comment applies to line 108, please describe the differeces in the homology between the proteins. For example, percent similar, percent identical, etc.
* The graph in Fig 2A is not easy to understand at first glance. Could a legend be added that explains that DF1, DF2, and DF3 are bound RNAs?
* Line 161-162 and Fig 2C: Given the enrichment of m6A in p-bodies, these data indicate that YTHDF KD stabilizes m6A transcripts preferentially, not necessarily p-body. However, an analyses comparing stability of m6A transcripts from the Depl vs Enr fractions could make this point.
* In Fig 2E and 2F, it would be helpful to add a label below or the left of "Depl" and "Enr" to state "P body".
* Fig S2E legend should indicate that cells were treated with siYTHDF1-3 (right?).

**Reviewer 2**

This manuscript by Zou et al. aims to shed further light on the function of YTHDF1-3 proteins in mammalian RNA biology. YTHDF proteins have been extensively studied over the past 10 years and are the prototypical epitranscriptomic reader proteins, which recognize m6A-modified RNA through their YTH domain. Previous work by the authors and by others has established a role for YTHDF proteins in RNA metabolism and limited studies have also shown effects on translation, however it remains unknown how the 3 YTHDF proteins work in concert to facilitate epitranscriptomic gene regulation. Towards this end, Zaccara and Jaffrey (Cell, 181, 1582, 2020) proposed that all 3 proteins have redundant function and promote the degradation of m6A modified transcripts, but do not have a role in translation. The current work seeks to refute this model and establish that the 3 YTHDF proteins are indeed functionally distinct. My specific comments can be found below, with a summary paragraph at the very end.

The authors start by discussing perceived flaws in the analysis of Zaccara and Jaffrey. They focus on the fact that Zaccara and Jaffrey analyzed the effects of individual YTHDF protein knockdown on all m6A-modified transcripts rather than only on the specific targets of each YTHDF protein (identified by CLIP analysis). While this seems like a reasonable argument, what the authors fail to mention is that Zaccara and Jaffrey analyzed multiple YTHDF1/2/3 CLIP datasets and concluded that all 3 proteins bind to the same m6A RNA targets, which is also in line with structural and biochemical data investigating the interactions of purified YTH domains with m6A oligonucleotides, and the overall high sequence conservation among these protein domains. This calls into question the idea that YTHDF proteins have unique RNA targets (as shown in Fig. 1a for YTHDF1/2). Regarding the second criticism that Zaccara and Jaffrey only analyzed actively translating genes, it is unclear as to what portion of the previous paper they are referring to; the claim that YTHDF1 does not affect translation in that paper was made based upon ribosome profiling experiments performed as well as by reanalysis of previous YTHDF1 data which showed a lack of consistency among independent biological replicates (Wang et al. Cell 2015). The authors then show a reanalysis of the knockdown data (Fig. S1A) using all expressed genes rather than just the actively translated ones and claim that YTHDF1 and YTHDF3 knockdown does not stabilize RNA whereas YTHDF2 does; this is in line with the findings from Zaccara and Jaffrey which also showed that individual knockdown of YTHDF1 and YTHDF3 was compensated for by YTHDF2 resulting in little effect on RNA stability, further it does nothing to address whether YTHDF1/3 affect translation efficiency. Next, the authors claim they have reanalyzed the ribosome profiling data looking only at YTHDF1 or YTHDF2 unique transcripts, and see that YTHDF1 knockdown impacts the translation of YTHDF1 unique transcripts and YTHDF1/2 shared targets but not of YTHDF2 unique targets; again this analysis is suspect in light of the Zaccara and Jaffrey work that shows that there are no unique targets among YTHDF1/2/3, and this issue (assignment of YTHDF1-3 targets based on CLIP data) is not addressed in the current work.

Next, the authors shift to investigating the disordered C-terminal domains and protein interaction partners of YTHDF1-3. Fig 1e shows the alignment of the disordered domains, which appear to be very similar, as was commented on by Zaccara and Jaffrey (60% amino acid identify and 70% amino acid similarity). The authors comment that the domains "differ greatly" however this seems like an exaggeration. They show some electron microscopy data on what I think is purified YTHDF1-3 which demonstrates that YTHDF2 forms fibrils whereas YTHDF1 and YTHDF3 do not and use this to support the idea that the disordered domains behave differently. While this is certainly possible, the data is far from rigorous - there is no description of protein purification in the Methods and no proof of purity. The observed fibrils (which only form after 2 week incubation, calling into question their biological relevance) could easily be a contaminant in the protein prep. In Fig. 1g, the authors analyze publicly available protein localization data to claim that YTHDF1/3 shows similar subcellular localization as eukaryotic translation initiation factors (EIF) whereas YTHDF2 colocalizes better with RNA degradation machinery (CNOT). This is interesting but also far from conclusive as the data does not reflect protein-protein interactions but only similar localization. In contrast, Zaccara and Jaffrey analyzed Bio-ID proteomic data (which is a better surrogate for protein interactions) in their paper to show that all 3 YTHDF proteins interact with CNOT, whereas EIF proteins were only picked up as weak hits and were present for all 3 YTHDF proteins.

In the final paragraph of this section, the authors claim that YTHDF1 is more abundant in HeLa cells than YTHDF2 while YTHDF2 is more abundant in HEK cells (Fig. S1d). Based on the western blot data presented (which is also quite noisy), this is not accurate. Levels of YTHDF1-3 appear to be comparable in HeLa cells.

In the second part of the paper, there is a shift in focusing on m6A-YTH interactions biology to looking at the role of YTHDF proteins in other cellular processes. The authors perform RNAseq with spike-in controls to show that YTHDF knockdown results in global mRNA stabilization irrespective of mRNA m6A status. They then show that P-body number is increased in triple YTHDF knockdown cells - the IF images in Fig. S2B need to be enlarged as it is very difficult to see the trends in P-body number. Also, how many cells were counted? There is no information on this.

Following up on the P-body phenotype, they perform RIP-seq on P-body associated transcripts and show that both unmethylated and m6A transcripts are found and are equally stabilized in the YTHDF1-3 knockdown condition and that stabilization depends upon the P-body protein DDX6.

In summary, this paper reads in two parts. The first part primarily deals with the reanalysis of available RNA expression, protein translation, and protein localization data in response to the Zaccara and Jaffrey Cell paper. I did not find this to be a particularly compelling response (especially in light of the rigorous data treatment in the Zaccara paper) for the reasons described above, and therefore and only serves to further muddy the waters without providing much resolution. In the second part, the authors connect YTHDF proteins to P-body biology. I think this is quite interesting and novel, but unfortunately not very well developed. I would support a significantly revised manuscript that focused primarily on the m6A-independent roles of YTHDF proteins and connections to P bodies.

**Authors’ response to reviewers**

Reviewer 1:

Comment: The YTHDF m6A reader proteins (YTHDF1, 2, and 3) have all been shown previously to bind m6A-containing mRNA. While each protein has been shown to have different effects on regulating RNA biology, including translation for DF1 and RNA decay for DF2, and on specific phenotypes (e.g. in KO mouse models), a recent manuscript (Zaccara & Jaffrey, PMID: 32492408) has suggested that the three proteins are redundant for each other and that they only regulate mRNA degradation and cellular differentiation. This was based in part on the finding that depletion of three results in increased mRNA abundance, with no effects on translation. This manuscript attempts to show that in fact these proteins, specifically DF1 and DF2, do have specific, individual roles in RNA biology and they are not redundant. Intriguingly, the manuscript presents data that suggests that loss of all three of the DF proteins, but not individual, actually results in increased formation of P bodies where
m6A RNA is enriched and stabilized, and that might explain previous observations that showed that loss of all three proteins phenocopies loss of DF2. While the study is systematic in re-analyzing previous data to show that YTHDF proteins do in fact have individual functions, there are instances where rationale was not clear and certain controls and interpretations needed to be included, revised, or explained more clearly (see comments below). However, the analyses do support a conclusion that YTHDF proteins have individual function to regulated specific subsets of RNA (YTHDF CLIP targets) and previous analyses of larger subsets of RNA (total or actively translated) obscured these functions. Thus, overall, this is an important study as it attempts to clarify the differences between published studies, and it will be important for the RNA biology field.

Response: We thank the reviewer for the encouraging comments and appreciation of this work. We have made revisions to address this reviewer’s comments as listed below:

* The text of this manuscript is written in a very antagonistic and judgmental way. See Line 55, "two flaws", Line 63, "not germane" Line 81, not appropriate; Line 84, "skewed choices". The manuscript would be more powerful if the work was presented in a non-judgemental fashion. Just say what was seen in the previous work, and then say what you did and why the results differ. For example, rather than saying "It is not germane to analyze the effects of YTHDF1 knockdown on translation or stabilization of ~60% of the transcriptome as potential targets of YTHDF1…" one could say, "While a previous study examined the effects of YTHDF1 knockdown on translation or stabilization of ~60% of the transcriptome…., as we know that the protein only binds ~10% of the transcriptome, we chose to specifically study the effects of YTHDF1 on its bound transcripts"… etc .

We thank the reviewer for the suggestions and we have re-phrased all of the judgmental statements identified in the current manuscript. Examples include: Line 57, “We found two flows in the analysis of Zaccara et al.” to “We identified two key methodological choices by Zaccara et al. in their analyses which differ from our methodology and likely contribute to divergent conclusions”; Line 65, “It is not germane to analyze the effects of YTHDF1 knockdown….. may be indirect” to “While Zaccara et al. examined the effects of YTHDF1 knockdown on translation or stability of ~60% of the transcriptome, YTHDF1 mainly binds only ~10% of the transcriptome. Thus, the effects of YTHDF1 knockdown on other m6A-modified mRNA could be indirect”; Line 85, “is not appropriate” to “may lead to incorporation of indirect effects in the analysis”; Line 84, “Thus, the claim that YTHDF proteins do not affect….experimental setup and analysis” is deleted now. Thank you for this suggestion.

* In Figure 1, data is presented on an m6A target centric (which m6A-modified mRNAs are regulate by DF1/DF2) vs RBP target centric approach (only looking at RNAs known to be bound by DF proteins). While the text clearly stated the limitations of an m6A-only approach, it wasn't obvious why the analyses performed were not filtered for transcripts with both m6A and RBP peaks from sequencing (m6A and RBP target centric approach). This could be even more informative.

We thank the reviewer for the insight, and we have performed the following analysis to examine YTHDF1,2,3 targets while filtering for m6A-modified transcripts. The conclusions are exactly the same as we got with RBP-centric approach in Fig. 1c-1d. YTHDF1 knockdown does not affect mRNA abundance, and only causes decreased translation efficiency of YTHDF1 targets. YTHDF2 knockdown only stabilizes YTHDF2 targets. Based on several references, analyzing the effects of YTHDF proteins based on RBP target information alongside filtering by m6A methylation status provides a better estimate of the physiological RNA substrates (Shi et al., PMID: 30401835, Han et al., PMID: 30728504). In the original draft, we tried to separate the RBP target-centric approach from m6A centric approach so we did not combine them. We have now included the results from the combined m6A-RBP-centric analysis in the supplementary figure S1d and added the following text in the manuscript: “Refining YTHDF targets to m6A-modified mRNA leads to the same conclusion (Additional data file 1: Fig. S1d).” (line 99).


* In Fig. 1A-1B, the numbers in the Venn diagrams vs those described in the text were not always clear. It would be helpful to label the figures differently. For example,

We thank the reviewer for the suggestions to make our manuscript easier to read. We have updated our Fig. 1A-1B in the revised manuscript. Regarding each specific questions raised by this reviewer, we are including our responses below:

♣ Fig. 1a: The text describes 4424 transcripts analyzed in Zaccara et al vs the 753 high confidence transcripts bound by YTHDF1, but it is unclear where this number comes from as the DF1 target numbers are 432+259+321=not 753.

We apologize we did not make this clear. The number of DF1 targets are 432+259+321+280=1292. However, only 432+321=753 of them are actively translated (overlapping with “Analyzed transcripts”) so they are used in the analysis of translation efficiency. These 753 are the analyzed DF1 targets in Fig. 1c and 1d. To clarify for the readers, we have replaced text “Analyzed transcripts” in Fig. 1a with text “Translating”. Also we have updated the entire Fig. 1a into an UpSet plot to make it easier to read.

♣ The overlaps and comparisons are difficult to follow in the text vs figure (referencing 7105 and 6814 when those are not in the diagrams without adding the numbers). Consider revising the text or figures (perhaps upset plots would help convey the points).

Per reviewer suggestions we have updated our Fig. 1a with an UpSet plot and highlighted that most analyzed genes in the previous work were not YTHDF targets.

♣ Figure 1B doesn't really make sense and the pictures aren't helpful. Perhaps just make a venn diagram with a legend.

This is a very good suggestion. We have updated Fig. 1b with a Venn diagram showing the number of genes analyzed in the previous work and this work.

* The data showing that purified YTHDF2 protein might make fibrils is potentially interesting, however some control data is missing. For example, how pure are the protein preps of each? I didn't see a Coomassie gel, etc. If each one has slightly different purification purities, this could explain the results. Also, it seems like the same time course (fresh, 2 weeks, 4 weeks) should be showing for the other YTHDF proteins to aid in comparison. Finally, while I do think this could get at the fact that the different proteins have different "natures", I'm not sure how this is related at all to their function, and this should be addressed.

We thank the reviewer for the suggestions. We have included the quality checks of these protein preps in figure S1e. And we removed the electron microscopy results for purified low complexity domains of YTHDF1,2 and 3 with prolonged incubation as the fresh protein preps already form distinct structures. We have also performed additional ThT fluorescence assays to study polymer formation of YTHDF proteins (Hudson et al., PMID: 19754881). For ThT assays, all fresh protein preps were denatured by 6M guanidine buffer to make sure proteins were in the monomer state. Then the proteins were diluted into native buffer and ThT fluorescence was monitored to follow polymer formation. YTHDF proteins show distinct polymer formation, which supports the conclusion that YTHDF proteins are different (Reviewer only Figure 1). The higher-order structure of proteins dictates their interaction with other biomolecules, and usually, determine their biological functions. More specifically, in the context of phase-separation, which is a critical feature of P-bodies we discussed later in this manuscript, the morphology of protein aggregates observed under EM is widely utilized as an estimate of their phase-separation abilities (Zhou et al., PMID: 35771920, Patel et al., PMID: 26317470).
We are continuing to investigate how the sequence differences may lead to structural changes and eventually distinct functional outcomes of these proteins. We added to the main text in line 122 the following sentences: “Proteins with low complexity domains may condense with different protein partners based on their intrinsic aggregate forming properties. RBPs in distinct RNP granules are known to have varied functional outcomes and bind different RNA pools.”

Reviewer only Figure 1: ThT assay showing the different polymer formation dynamics and kinetics of YTHDF protein LCDs.

* For the data comparisons of the YTHDF proteomics shown in Figure 1G and Supplemental Figure 1B, it's unclear why this analysis led to a different result than previous analyses. A more detailed method for analyses of the publicly available proteomics data is required, as well as a discussion that compares the analyses methods used by this paper and the Zaccara paper and describes possible discrepancies. It's not helpful to the field to just say the other paper is wrong.

We are using the same published dataset as the Zaccara paper (Zaccara & Jaffrey, PMID: 32492408). This study (Youn et al., PMID: 29395067) performed Bio-ID by fusing BirA* to the N-terminus or C-terminus of the bait protein, in our case, YTHDF1,2 and 3. The public dataset provided data generated with BirA* fusions of YTHDF1-C, YTHDF2-N, YTHDF2-C, YTHDF3-N, YTHDF3-C, respectively but not YTHDF1-N. To make the analysis consistent, we compared data generated from YTHDF1-C, YTHDF2-C and YTHDF3-C fusions. However, Zaccara et al. compared results from YTHDF1-C, YTHDF2-N and YTHDF3-N fusions, which compared C-terminal BirA* fusion of YTHDF1 with N-terminal BirA* fusions of YTHDF2,3, while we compared results from all C-terminal BirA* fusions of YTHDF1,2,3. We have now clearly described in the main text the difference in line 135.


* The data shown in Figure S1d are not convincing enough to show for the relative abundances of these proteins between the cell lines tested, in particular because the loading control GAPDH is not even in the replicates. A total protein stain should be done as the loading control, and a linear range of protein should be used, with purified protein as a control (e.g. 5ug, 20ug, 50ug protein).

We have added figure S1h which includes a more thorough analysis of HeLa and HEK293T lysates with the controls requested by the reviewer. We loaded a fixed amount (1 ng) of purified GST-tagged YTHDFs and serially diluted HeLa or HEK293T whole-cell lysates. We quantified individual lanes and used a 2-order polynominal regression to calculate the relative weight of YTHDF proteins in whole cell lysates (weight/weight). Consistent with our previous observation, we found that YTHDF1 and YTHDF3, which promote mRNA translation, are more abundant in HeLa cells. YTHDF2, which facilitates mRNA decay, is more abundant in HEK293T cells. We have adjusted the corresponding text in the manuscript.

* The quantification of the number of p bodies under different conditions (see Supp Fig 2B) doesn't seem right. When I look at the representative figures, it appears that siDF1 has a large increase in the number of P bodies as compared to siMETTL3. Thus, its unclear how this data led to the results shown in Fig 2B. Further, without having a cytoplasmic marker (or bright-field) markers, one can't examine whether the green dot for the p body is in a cell or just background. Finally, it is really hard to see the p body staining, and some image processing that makes them more clear for the figures would be helpful.

We apologize for the lack of clarity. We have enlarged the figures and updated supplementary Fig. 2b to make the results clearer for readers. We have also included number of cells analyzed under each condition. In addition, we have included one representative image showing bright-field (DIC) for cell margins below. We can see that DCP1A (P-body marker) signals localize exclusively within cells. Moreover, knockdown of DDX6 caused a significant decrease of DCP1A foci in cells (figure S2e) justifying our labeling strategy and the specificity of the DCP1A signals.

Figure 2: Representative images of P-body imaging (DCP1A) showing the signals exclusively localized to the cytosol.

* The last paragraph of the results was confusing and difficult to read, in particular the figure legends and data didn't always seem to match what was said in the text.

We apologize for the lack of clarity. We have rephrased the last paragraph of the results and included results with DDX6 overexpression. We aimed to show that P-body dynamics are important for the global mRNA stabilization after YTHDF triple knockdown.


* While the conclusion that YTHDF1-3 dependent stabilization of RNA is P-body, not m6A, -dependent is supported, it is not clear if P-body formation is sufficient for the phenotype. The text does state "that the global stabilization of mRNA after depletion of all YTHDF proteins is a result of increased P-body formation and is not strictly m6A dependent," however, an important experiment to support this claim would be to induce P-body formation with a stimuli other that YTHDF knockdown and observe RNA stability, perhaps over-expression of DDX6?

We thank this reviewer for the constructive suggestion. We have induced P-body formation with over-expression of DDX6 and performed spike-in normalized RNA-seq. We showed that DDX6 over-expression caused increased P-body formation in HeLa cells by DCP1A staining (Supplementary fig. 2e). Also, spike-in normalized RNA-seq showed that DDX6 overexpression caused m6A-independent RNA stabilization (Supplementary fig. 2f, left panel) and that triple knockdown of YTHDFs exaggerated the effects of DDX6 overexpression (Supplementary fig. 2f, right panel).

Minor comments:
* In line 31 of background, YTHDF1 function is described, but no detail on known function of others. A short description/summary would be helpful.
We have added the following text to describe background of other YTHDF proteins, which is the main subject of this manuscript: “YTHDF2 accelerates the decay of its mRNA targets via recruiting the CCR4-NOT deadenylation complex. YTHDF3 facilitates translation and accelerates decay of its mRNA targets”.

* Fig. S1A: YTHDF3-dependent decreases are not obvious in these data. Text should be revied to only include YTHDF1-dependent changes
* Fig. S1A: YTHDF3-dependent changes in translated genes appear to be more significant than expressed genes alone (according to indicated stats). This is contrary to the point made in the text. Please fix figures if needed or revise test to match data.
We have updated the text in the manuscript from “… tends to decrease more upon YTHDF1 or YTHDF3 knockdown…” to “…tends to decrease more upon YTHDF1 knockdown…”

* Fig 1C-D: To better understand the difference between the conclusion of the authors and previously held models, please include differences in TE and abundance upon YTHDF1 or 2 knockdown when the original set 4424 transcripts is analyzed rather than exclusively YTHDF1 or 2 targets (compare with m6A data)
We thank this reviewer for the constructive suggestion. We are analyzing the same datasets as the report (Zaccara and Jaffrey, PMID: PMC7508256). Results of analysis of 4,424 m6A-modified RNA can be found in Figure 3 and Figure 4 of the original publication. Analyzing 4,424 RNA diminished the effects of YTHDF1 knockdown on translation efficiency of YTHDF1 targets. We have added the following text in the manuscript to better explain the results: “Analyzing all m6A-modified actively translated genes led to the conclusion that they do not affect mRNA translation”.

* Line 106: Please described how the YTHDF proteins differ in their low-complexity domains, rather than saying they differ "greatly"; Similar comment applies to line 108, please describe the differeces in the homology between the proteins. For example, percent similar, percent identical, etc.
We have quantified the amino acid similarities of YTHDF proteins with the BLOSUM62 algorithm individually either within the low complexity domain (LCD) or for full-length proteins. We have included the results in Fig. 1f. Our results show that similarities between YTHDF proteins are ~50% in their LCDs while the full-length proteins are more similar, with a ~65% score. We have also added the following text in the manuscript: “The similarity calculations between YTHDF proteins in their low-complexity domains (LCDs) are within the 49%-52% range while those for full-length proteins are 58%-69%, indicating that they mainly differ in their LCDs (Fig. 1f).”

* The graph in Fig 2A is not easy to understand at first glance. Could a legend be added that explains that DF1, DF2, and DF3 are bound RNAs?
We have added a text legend “RNA bound (or modified) by” in Fig. 2a.

* Line 161-162 and Fig 2C: Given the enrichment of m6A in p-bodies, these data indicate that YTHDF KD stabilizes m6A transcripts preferentially, not necessarily p-body. However, an analyses comparing stability of m6A transcripts from the Depl vs Enr fractions could make this point.
We thank this reviewer for the comment. We have added a direct comparison of the m6A-modified transcripts from the Depl vs Enr fractions. We have also added the following text: “Moreover, the m6A-modified transcripts in the P-body-enriched fraction are more stabilized after YTHDF1-3 triple knockdown (Fig. 2c).”

* In Fig 2E and 2F, it would be helpful to add a label below or the left of "Depl" and "Enr" to state "P body".
We have added the text label as suggested in Fig. 2e and 3f.

* Fig S2E legend should indicate that cells were treated with siYTHDF1-3 (right?).
We apologize we did not make this clear. Figure S2e was a quality check showing that DDX6 knockdown caused significantly decreased P-body formations in cells. Those cells were not treated with siYTHDF1-3. We have updated Figure S2e with also overexpression of DDX6.

Reviewer #2:
This manuscript by Zou et al. aims to shed further light on the function of YTHDF1-3 proteins in mammalian RNA biology. YTHDF proteins have been extensively studied over the past 10 years and are the prototypical epitranscriptomic reader proteins, which recognize m6A-modified RNA through their YTH domain. Previous work by the authors and by others has established a role for YTHDF proteins in RNA metabolism and limited studies have also shown effects on translation, however it remains unknown how the 3 YTHDF proteins work in concert to facilitate epitranscriptomic gene regulation. Towards this end, Zaccara and Jaffrey (Cell, 181, 1582, 2020) proposed that all 3 proteins have redundant function and promote the degradation of m6A modified transcripts, but do not have a role in translation. The current work seeks to refute this model and establish that the 3 YTHDF proteins are indeed functionally distinct. My specific comments can be found below, with a summary paragraph at the very end.

Response: We thank the reviewer for the appreciation of the work aiming at clarifying the functions of YTHDF proteins. We want to state that there are many references indicating a role of YTHDF1 and 3 in promoting mRNA translation from other laboratories (including but not limited to: Chang et al., PMID: 33125861, Li et al., PMID: 28106076, Zhang et al., PMID: 30591559, Somasekharan et al., PMID: 34939643. Kastan et al., PMID: 33849973, Zong et al., PMID: 33999206, Yao et al., PMID: 35319018, Pi et al., PMID: 32788173).

The authors start by discussing perceived flaws in the analysis of Zaccara and Jaffrey. They focus on the fact that Zaccara and Jaffrey analyzed the effects of individual YTHDF protein knockdown on all m6A-modified transcripts rather than only on the specific targets of each YTHDF protein (identified by CLIP analysis). While this seems like a reasonable argument, what the authors fail to mention is that Zaccara and Jaffrey analyzed multiple YTHDF1/2/3 CLIP datasets and concluded that all 3 proteins bind to the same m6A RNA targets, which is also in line with structural and biochemical data investigating the interactions of purified YTH domains with m6A oligonucleotides, and the overall high sequence conservation among these protein domains. This calls into question the idea that YTHDF proteins have unique RNA targets (as shown in Fig. 1a for YTHDF1/2).

We thank the reviewer for raising these concerns and we would like to address those in the following aspects:
o The YTH domains of YTHDF proteins do have significant similarities, but are not the same. In support of this, we have recently developed small-molecule inhibitors targeting YTHDF1 or YTHDF2. Our YTHDF1 inhibitor shows a ~20 fold selectivity in binding the YTH domain of YTHDF1 over YTHDF2 (unpublished result). A docking model shows that several amino acids within the YTH domain of YTHDF1 (N411, N504, S505, R506 and D507) are critical for the selectivity. Our YTHDF2 inhibitor shows an ~8 fold selectivity in binding the YTH domain of YTHDF2 over YTHDF1 (Unpublished result).
o The N-terminus of the YTHDF proteins are different. We have quantified the amino acid similarities of YTHDF proteins with the BLOSUM62 algorithm individually within the low complexity domain (LCD) or full-length proteins. We have included the results in Fig. 1f. Our results show that similarities between YTHDF proteins are ~50% in their LCDs while the full length proteins are more similar, with a ~65% score. We have also added the following text in the manuscript: “The similarity calculations between YTHDF proteins in their low-complexity domains (LCDs) are within the 49%-52% range while those for full-length proteins are 58%-69%, indicating that they mainly differ in their LCDs (Fig. 1f).” This part has now been thoroughly described in the updated Fig. 1e-1g.
o PAR-CLIP can identify individual, different RNA targets of YTHDF proteins. Zaccara and Jaffrey argued that YTHDF proteins have different targets by comparing the reads coverage. However, PAR-CLIP is known to be hampered by co-immunoprecipitation and peaks should be called by taking mutation numbers into consideration. T-C mutations are caused by direct binding of protein on incorporated 4-thiouridine in mRNA. We have provided a supplementary figure (Figure 3 below, with panels a and b also shown in supplementary figure 1b and 1c) analyzing the mutations in PAR-CLIP experiments. In (a) and (b), we showed that YTHDF1 and YTHDF2 have different mutation profiles in PAR-CLIP experiments on the whole transcriptome. In (c), we show that the defined YTHDF1 and YTHDF2 targets show higher mutation rates than all other targets in their corresponding PAR-CLIP experiments, individually. Thus, many YTHDF1 or YTHDF2 unique targets are specific to each protein. In (d), we showed that our previous method to combine RIP+CLIP experiments helps to identify more confident RNA targets of YTHDF1/2.
o

Figure 3: Mutation profiles of YTHDF1 and YTHDF2 targets. (a), Mutation numbers of each transcript in YTHDF1/YTHDF2 PAR-CLIP experiments. (b), Heatmap showing the mutation numbers of several unique targets of YTHDF1 or YTHDF2. (c), Boxplots showing the mutation numbers of mRNA grouped as DF1, DF2 or both targets in YTHDF1/YTHDF2 PAR-CLIP experiments. (d), Boxplots showing the mutation numbers of mRNA grouped by CLIP and RIP experiments in YTHDF1 or YTHDF2 PAR-CLIP experiments.

Regarding the second criticism that Zaccara and Jaffrey only analyzed actively translating genes, it is unclear as to what portion of the previous paper they are referring to; the claim that YTHDF1 does not affect translation in that paper was made based upon ribosome profiling experiments performed as well as by reanalysis of previous YTHDF1 data which showed a lack of consistency among independent biological replicates (Wang et al. Cell 2015).

As far as we can tell based on a thorough and detailed examination of their manuscript, figures, supplemental files, and public datasets, Zaccara and Jaffrey used actively translation genes in all their analyses, including but not limited to translation efficiency and RNA abundance. The claim that YTHDF1 does not affect translation is based on m6A-centric analyses and that took ~60% of the transcriptome into consideration. We have updated the Fig. 1a to make this clear to the audience. More than 3,000 genes out of 4,424 genes analyzed by Zaccara and Jaffrey were not bound by any YTHDF protein. Additionally, Zhang et al. provide important background in Fig. 4d of this Nat. Genetics paper (https://www.nature.com/articles/s41588-020-0644-z) (reproduced blow). While YTHDF1 promotes translation of m6A-methylated transcripts, there are a number of non-YTHDF RBPs that bind m6A-methylated transcript and suppress their translation. What Zaccara and Jaffrey did was to average the whole spectrum of m6A methylated transcripts instead of analyzing transcripts bound by each individual RBP for their functions, which may lead to incorporation of indirect effects in the analysis. We have also shown that YTHDF1,2 and 3 have distinct targets above.


The authors then show a reanalysis of the knockdown data (Fig. S1A) using all expressed genes rather than just the actively translated ones and claim that YTHDF1 and YTHDF3 knockdown does not stabilize RNA whereas YTHDF2 does; this is in line with the findings from Zaccara and Jaffrey which also showed that individual knockdown of YTHDF1 and YTHDF3 was compensated for by YTHDF2 resulting in little effect on RNA stability, further it does nothing to address whether YTHDF1/3 affect translation efficiency.

YTHDF1 and YTHDF3 should not be compensated by YTHDF2 in HeLa cells as YTHDF3 is more abundant than YTHDF2 in HeLa cells. We have shown the relative protein levels in Fig. S1D in the original submission and have provided an updated quantification with purified YTHDF proteins as controls. We have added supplementary figure S1e conducting a more thorough analysis with HeLa and HEK293T lysates with the controls this reviewer asked for. We loaded fixed amount (1 ng) of purified GST-tagged YTHDFs and serially diluted HeLa or HEK293T whole-cell lysates. We quantified individual lanes and used a 2-order polynominal regression to calculate the relative weight of YTHDF proteins in whole cell lysates (weight/weight). Consistent with our previous result, we found that YTHDF1 and YTHDF3, which promotes mRNA translation, are more abundant in HeLa. YTHDF2, which facilitates mRNA decay, is more abundant in HEK293T cells. We have adjusted the corresponding texts in the manuscript. Our responses to previous comments from reviewers 1 and 2 have already addressed whether YTHDF1 and 3 affect translation efficiency, with data not only from our own laboratory but also the broad community.

Next, the authors claim they have reanalyzed the ribosome profiling data looking only at YTHDF1 or YTHDF2 unique transcripts, and see that YTHDF1 knockdown impacts the translation of YTHDF1 unique transcripts and YTHDF1/2 shared targets but not of YTHDF2 unique targets; again this analysis is suspect in light of the Zaccara and Jaffrey work that shows that there are no unique targets among YTHDF1/2/3, and this issue (assignment of YTHDF1-3 targets based on CLIP data) is not addressed in the current work.

We have addressed this concern above and our analysis showed that YTHDF1,2 and 3 have unique targets (see Figure 3 of this response letter).

Next, the authors shift to investigating the disordered C-terminal domains and protein interaction partners of YTHDF1-3. Fig 1e shows the alignment of the disordered domains, which appear to be very similar, as was commented on by Zaccara and Jaffrey (60% amino acid identify and 70% amino acid similarity). The authors comment that the domains "differ greatly" however this seems like an exaggeration. They show some electron microscopy data on what I think is purified YTHDF1-3 which demonstrates that YTHDF2 forms fibrils whereas YTHDF1 and YTHDF3 do not and use this to support the idea that the disordered domains behave differently. While this is certainly possible, the data is far from rigorous - there is no description of protein purification in the Methods and no proof of purity. The observed fibrils (which only form after 2 week incubation, calling into question their biological relevance) could easily be a contaminant in the protein prep.

We have included the quality checks of these protein preps in figure S1b. And we included the electron microscopy results for purified low complexity domains of YTHDF1,2 and 3 with prolonged incubation to clarify our findings. We thank this reviewer for appreciating that YTHDF1,2 and 3 have different natures. The structures of proteins dictate their interaction with other biomolecules, and usually, determine their biological functions. More specifically, in the context of phase-separation, which is a critical feature of P-bodies we discussed later in this manuscript, the morphology of protein aggregates observed under EM is widely utilized as an estimate of their phase-separation abilities (Zhou et al., PMID: 35771920, Patel et al., PMID: 26317470). We have added the following information to the main text: “Proteins with low complexity domains may condensate with different protein partners based on their intrinsic aggregate forming properties. RBPs in distinct RNP granules are known to have varied functional outcomes.”

In Fig. 1g, the authors analyze publicly available protein localization data to claim that YTHDF1/3 shows similar subcellular localization as eukaryotic translation initiation factors (EIF) whereas YTHDF2 colocalizes better with RNA degradation machinery (CNOT). This is interesting but also far from conclusive as the data does not reflect protein-protein interactions but only similar localization. In contrast, Zaccara and Jaffrey analyzed Bio-ID proteomic data (which is a better surrogate for protein interactions) in their paper to show that all 3 YTHDF proteins interact with CNOT, whereas EIF proteins were only picked up as weak hits and were present for all 3 YTHDF proteins.

We are using the same published dataset as the Zaccara paper (Zaccara & Jaffrey, PMID: 32492408). This study (Youn et al., PMID: 29395067) performed Bio-ID by fusing BirA* to the N-terminus or C-terminus of the bait protein, in our case, data for YTHDF1,2, and 3 were selected. The public dataset provided data generated with BirA* fusions of YTHDF1-C, YTHDF2-N, YTHDF2-C, YTHDF3-N, YTHDF3-C, respectively. To make the analysis consistent, we compared data generated with YTHDF1-C, YTHDF2-C and YTHDF3-C BirA* fusions. However, Zaccara paper compared BirA* fusions of YTHDF1-C, YTHDF2-N and YTHDF3-N, respectively, which compared results from the C-terminal BirA* fusion of YTHDF1 with those of N-terminal BirA* fusions of YTHDF2,3.

We hope the reviewer would agree with our approach that comparing results from all C-terminal BirA* fusions of YTHDF proteins is more consistent. We have now clearly described in the main text the difference in line 135.

In the final paragraph of this section, the authors claim that YTHDF1 is more abundant in HeLa cells than YTHDF2 while YTHDF2 is more abundant in HEK cells (Fig. S1d). Based on the western blot data presented (which is also quite noisy), this is not accurate. Levels of YTHDF1-3 appear to be comparable in HeLa cells.

We have shown the relative protein levels in Fig. S1e in the original submission. We have added supplementary figure S1e conducting a more thorough analysis with HeLa and HEK293T lysates with the controls this reviewer asked for. We loaded fixed amount (1 ng) of purified GST-tagged YTHDFs and serial diluted HeLa or HEK293T whole-cell lysates. We quantified individual lanes and used a 2-order polynominal regression to calculate the relative weight of YTHDF proteins in whole cell lysates (weight/weight). Consistent with our previous result, we found that YTHDF1 and YTHDF3, which promotes mRNA translation, are more abundant in HeLa. YTHDF2, which facilitates mRNA decay, is more abundant in HEK293T cells. We have adjusted the corresponding text in the manuscript. This also suggests that YTHDF2 is not the most abundant YTHDF in HeLa or HEK293T cells, and thus cannot compensate for YTHDF1 or YTHDF3 in individual knockdowns.


In the second part of the paper, there is a shift in focusing on m6A-YTH interactions biology to looking at the role of YTHDF proteins in other cellular processes. The authors perform RNAseq with spike-in controls to show that YTHDF knockdown results in global mRNA stabilization irrespective of mRNA m6A status. They then show that P-body number is increased in triple YTHDF knockdown cells - the IF images in Fig. S2B need to be enlarged as it is very difficult to see the trends in P-body number. Also, how many cells were counted? There is no information on this.

We apologize for the lack of clarity. We have enlarged the figures and updated supplementary Fig. 2b to make the results more apparent for readers. We have also included the number of cells analyzed under each condition. In addition, we have included one representative image showing bright-field (DIC) for cell margins below. We can see that DCP1A (P-body marker) signals localize exclusively within cells. Moreover, knockdown of DDX6 caused a significant decrease of DCP1A foci in cells (Figure S2e), justifying our labeling strategy and the specificity of the DCP1A signals.


Following up on the P-body phenotype, they perform RIP-seq on P-body associated transcripts and show that both unmethylated and m6A transcripts are found and are equally stabilized in the YTHDF1-3 knockdown condition and that stabilization depends upon the P-body protein DDX6. In summary, this paper reads in two parts. The first part primarily deals with the reanalysis of available RNA expression, protein translation, and protein localization data in response to the Zaccara and Jaffrey Cell paper. I did not find this to be a particularly compelling response (especially in light of the rigorous data treatment in the Zaccara paper) for the reasons described above, and therefore and only serves to further muddy the waters without providing much resolution. In the second part, the authors connect YTHDF proteins to P-body biology. I think this is quite interesting and novel, but unfortunately not very well developed. I would support a significantly revised manuscript that focused primarily on the m6A-independent roles of YTHDF proteins and connections to P bodies.

We have addressed the concerns related to the first comment above. We showed that YTHDF proteins have different sequences, form different structures and possess distinct functions. How does triple knockdown cause the most significant RNA stabilization? Here we provide one pathway we believe is an important cause. Other RBPs could be involved as well. We want to thank this reviewer for appreciating the importance of our findings and for their questions that help us clarify our work. Indeed, it requires careful reading and comparison to see subtle but functionally significant differences between the two works.

**Second round of review**

**Reviewer 1**

This revision presents a clearer and more well-explained analysis of the data, along with some new analyses and experiments that significantly strengthens their conclusions - that YTHDF proteins can have different targets and thus functions in the cells and that loss of all three DF proteins results in changes to P bodies that stabilize global mRNA levels of P-body enriched transcripts.

For this reviewer, the strengths include analyzing the PAR-CLIP mutation numbers induced by YTHDF cross-linking as a better readout for binding showing that they do indeed have unique targets; the fact that more than 3000/4424 m6A-marked genes analyzed by the other papers were not bound by any YTHDF protein, suggesting that these 3000 genes are masking individiual YTHDF affects; increased rigor in the experiments showing YTHDF protien expression in different cell types; increased description of the analysis of DF protein-BirA experiments; and clear data that shows depletion of all three DF proteins increases P body formation, which results in increased RNA abundance of many, not just m6A-containing, transcripts.

Minor comments
-Another paper was recently published which supports this work (PMID: 36302554). This papers uses YTHDF proteins fused to RNA editors (TRIBE/STAMP) to map multiple DF protein binding to the same mRNA. It finds that individual mRNAs can be bound by more than one DF protein over their lifetime and that there are DF1 unique targets. A discussion and/or citation of this manuscript would further strengthen the conclusions of this paper.
-The new supplementary Figure 2F was not present in the supplemental figures.

**Reviewer 2**

I commend the authors on this revised manuscript that does an excellent job of clarifying the key methodological distinctions between this work and prior work. The experiments with DDX6 KD/overexpression also strengthen the argument regarding PB-mediated stabilization of transcripts in the triple YTHDF KO cells. As such, I support publication in the current form with only one small point that should be addressed regarding analysis of the Bio-ID data -- can the authors analyze the proximity labeling with N-terminal BirA fusions of YTHDF1-3 and compare against the C-terminal fusions? I agree that mixing and matching comparisons with N-term/C-term labels seems odd, but the manuscript should comment on the affect of label orientation on the conclusions.

**Authors’ response to reviewers**

Reviewer 1:

Reviewer #1: This revision presents a clearer and more well-explained analysis of the data, along with some new analyses and experiments that significantly strengthens their conclusions - that YTHDF proteins can have different targets and thus functions in the cells and that loss of all three DF proteins results in changes to P bodies that stabilize global mRNA levels of P-body enriched transcripts.

For this reviewer, the strengths include analyzing the PAR-CLIP mutation numbers induced by YTHDF cross-linking as a better readout for binding showing that they do indeed have unique targets; the fact that more than 3000/4424 m6A-marked genes analyzed by the other papers were not bound by any YTHDF protein, suggesting that these 3000 genes are masking individiual YTHDF affects; increased rigor in the experiments showing YTHDF protien expression in different cell types; increased description of the analysis of DF protein-BirA experiments; and clear data that shows depletion of all three DF proteins increases P body formation, which results in increased RNA abundance of many, not just m6A-containing, transcripts.

Response: We thank this reviewer for the warm and constructive comments throughout the review process, and we sincerely hope this report could help clarify confusions in the YTHDF research.

Minor comments
-Another paper was recently published which supports this work (PMID: 36302554). This papers uses YTHDF proteins fused to RNA editors (TRIBE/STAMP) to map multiple DF protein binding to the same mRNA. It finds that individual mRNAs can be bound by more than one DF protein over their lifetime and that there are DF1 unique targets. A discussion and/or citation of this manuscript would further strengthen the conclusions of this paper.
Response: This is a very good suggestion. We have included the discussion of this paper on page 6 in the main text “Moreover, a recent report using individual YTHDF proteins fused to RNA editors (TRIBE/STAMP), respectively, to map mRNA binding by YTHDF proteins. This study showed that individual mRNAs could be bound by more than one YTHDF protein over their lifetime, and that there are YTHDF1 unique target mRNAs as also shown in our analysis [22].”

-The new supplementary Figure 2F was not present in the supplemental figures.

Response: We are sorry about the mistake. In the previous rebuttal letter, we mistakenly wrote Fig. S2f instead of Fig. 2f. This is the original text “Also, spike-in normalized RNA-seq showed that DDX6 overexpression caused m6A-independent RNA stabilization (Supplementary fig. 2f, left panel) and that triple knockdown of YTHDFs exaggerated the effects of DDX6 overexpression (Supplementary fig. 2f, right panel).” The contents referred to in the text were actually Fig. 2f. We have corrected this mistake in the main text and carefully checked all the figure labels.


Reviewer #2: I commend the authors on this revised manuscript that does an excellent job of clarifying the key methodological distinctions between this work and prior work. The experiments with DDX6 KD/overexpression also strengthen the argument regarding PB-mediated stabilization of transcripts in the triple YTHDF KO cells. As such, I support publication in the current form with only one small point that should be addressed regarding analysis of the Bio-ID data -- can the authors analyze the proximity labeling with N-terminal BirA fusions of YTHDF1-3 and compare against the C-terminal fusions? I agree that mixing and matching comparisons with N-term/C-term labels seems odd, but the manuscript should comment on the affect of label orientation on the conclusions.

Response: We thank the reviewer for the encouraging comment.
In the original paper that reported the Bio-ID datasets (PMID: 29395067). They discussed the data overlap between N-term/C-term fusions: “…To assess the degree of overlap in the recovery of high-confident preys between the N- and C-terminal fusions, we used the terminus with fewer preys as reference. The details of overlap are listed here: CELF1 – 82%, CNOT9 – 90%, FAM120C – 76%, FMR1 – 82%, FXR1 – 94%, FXR2 – 86%, KIAA0355 – 65%, KIF1B – 45%, MKRN2 – 69%, SDCCAG3 – 68%, SMG7 – 48%, STAU1 – 73%, TDRD3 – 80%, TNRC6A – 62%....” Thus, the degree of overlap for different proteins can be different. For YTHDF2 and YTHDF3, both N-term/C-term fusions are available, while for YTHDF1, only the C-term fusion was available so we chose do compare all YTHDFs with the C-term fusions, which was the most rigorous choice given data available.
We have added the following comments on the affect of label orientations on the conclusions in the manuscript: “The report constructed the Bio-ID database comparing the overlap between N-terminal fusion and C-terminal fusion of the same protein. The results showed overlap ratio varying between 45% to 92% [26]. Thus, using data obtained from YTHDF proteins with the BirA* fusion at the same terminus provides more appropriate comparison of their protein partners.”
